# Increased Cerebral Water Content in Hemodialysis Patients

**DOI:** 10.1371/journal.pone.0122188

**Published:** 2015-03-31

**Authors:** Kathrin Reetz, Zaheer Abbas, Ana Sofia Costa, Vincent Gras, Frances Tiffin-Richards, Shahram Mirzazade, Bernhard Holschbach, Rolf Dario Frank, Athina Vassiliadou, Thilo Krüger, Frank Eitner, Theresa Gross, Jörg Bernhard Schulz, Jürgen Floege, Nadim Jon Shah

**Affiliations:** 1 Department of Neurology, RWTH Aachen University Hospital, Germany; 2 Institute of Neuroscience and Medicine (INM-4), Research Centre Jülich GmbH, Jülich, Germany; 3 Jülich Aachen Research Alliance (JARA)—Translational Brain Medicine, Jülich and Aachen, Germany; 4 KfH Kuratorium für Dialyse und Nierentransplantation e.V., Stolberg, Germany; 5 Department of Internal Medicine, St.-Antonius-Hospital Eschweiler, Eschweiler, Germany; 6 Dialysezentrum Aachen, Praxis und Dialyse, Aachen, Germany; 7 Division of Nephrology and Clinical Immunology, RWTH Aachen University, Aachen, Germany; West China Hospital of Sichuan University, CHINA

## Abstract

Little information is available on the impact of hemodialysis on cerebral water homeostasis and its distribution in chronic kidney disease. We used a neuropsychological test battery, structural magnetic resonance imaging (MRI) and a novel technique for quantitative measurement of localized water content using 3T MRI to investigate ten hemodialysis patients (HD) on a dialysis-free day and after hemodialysis (2.4±2.2 hours), and a matched healthy control group with the same time interval. Neuropsychological testing revealed mainly attentional and executive cognitive dysfunction in HD. Voxel-based-morphometry showed only marginal alterations in the right inferior medial temporal lobe white matter in HD compared to controls. Marked increases in global brain water content were found in the white matter, specifically in parietal areas, in HD patients compared to controls. Although the global water content in the gray matter did not differ between the two groups, regional increases of brain water content in particular in parieto-temporal gray matter areas were observed in HD patients. No relevant brain hydration changes were revealed before and after hemodialysis. Whereas longer duration of dialysis vintage was associated with increased water content in parieto-temporal-occipital regions, lower intradialytic weight changes were negatively correlated with brain water content in these areas in HD patients. Worse cognitive performance on an attention task correlated with increased hydration in frontal white matter. In conclusion, long-term HD is associated with altered brain tissue water homeostasis mainly in parietal white matter regions, whereas the attentional domain in the cognitive dysfunction profile in HD could be linked to increased frontal white matter water content.

## Introduction

In chronic kidney disease (CKD) patients, cognitive impairment is highly prevalent and increases as kidney function worsens [1, 2]. In patients undergoing hemodialysis (HD), it markedly exceeds values observed in age-matched controls [3, 4]. Knowledge of cerebral changes in CKD, as well as the impact of HD on cognition and brain function is limited. Although the brain and the kidneys both show a similar vulnerability to vascular damage [5], their pathophysiological linkage is multifaceted. Both suffer damage from hypertension, diabetes, hypercholesterolemia, anemia and inflammatory changes [6–8] resulting in an increased prevalence of silent brain infarcts as well as microbleeds in CKD patients [8]. Besides cerebrovascular changes, a direct uremic neuronal toxicity supports additional neurodegenerative pathways [9]. Although the cerebrovascular aspect is also supported by the neuropsychological pattern of cognitive impairment in CKD patients, namely executive functions and psychomotor speed deficits [10], memory-relevant alterations have also been reported [11]. Furthermore, cognitive functions fluctuate over the course of the dialysis cycle and cognitive functioning is mostly vulnerable during the dialysis session [12]. This finding suggests that direct consequences of CKD such as overhydration and/or the uremic milieu may also affect cognitive functioning [13].

Non-invasive, quantitative mapping of water content in the *in vivo* human brain has demonstrated the ability to provide important disease-related parameters associated with focal or global change in tissue water homeostasis [14]. We used a recently validated magnetic resonance imaging (MRI) technique for quantitative water content measurement [15, 16] to investigate brain water changes in HD patients.

The aim of the study was to identify such changes in late stage CKD and possible variations during the dialysis cycle, as well as their relation to clinical disease-specific features using a recently validated whole-brain MRI sequence.

## Methods

### Participants

Ten patients undergoing hemodialysis in the greater Aachen area were recruited (for detailed characteristics of the study sample please see Table 1). The control group comprised ten healthy volunteers with no history of cardiovascular or kidney diseases matched for age, gender and education. Exclusion criteria for all study participants included contraindications for research MRI, severe sensory impairment, and previous history of neurological or psychiatric disease. All subjects provided written informed consent for participation in this study, which was approved by the local ethics committee of the RWTH Aachen University (EK 179/11) and conducted in accordance to the latest version of the Helsinki declaration.

**Table 1:** Demographic and clinical characteristics of the hemodialysis patients and healthy control groups.

Demographic and clinical variables	Hemodialysis patients	Healthy controls	*p*-value
	n = 10	n = 10	
Age, years	45.80 (12.67)	50.30 (14.44)	.47
Sex, % female	40	40	1
Education, years	12.00 (3.06)	14.00 (2.16)	.11
**CV risk factors**			
Diabetes	20%	none	.47
Hypertension	90%	20%	<.05
Hypercholesterolaemia	10%	none	1
Smoking, (py)	6.3 (10.2)	0	.24
BMI (km/m^2^)	24.7 (4.4)	23.6 (3.3)	.68
Cardiovascular Risk (SCORE)	1 (1.5)	-	-
Charlson Comorbidity Index (CCI)	3.1 (1.7)	.80 (1.1)	<.01
**Medication**			
Antihypertensives	90%	20%	<.05
Insulin	20%	none	.47
Thyroid drugs	30%	none	.21
Antipsychotics	20%	none	.47
Antidepressants	20%	none	.47
Anxiolytics	10%	none	1
Analgesics	10%	none	1
Levodopa	10%	none	1
*n* affecting cognition	30%	none	.32
**Primary cause of CKD**			
Diabetic nephropathy	10%	-	-
Glomerulonephritis and systemic diseases	20%	-	-
Polycystic kidney disease	20%	-	-
Other/Unknown	20%/30%	-	-
**CKD associated comorbidities**			
Renal anemia	80%	-	-
Secondary hyperparathyroidism	20%	-	-
Renovascular hypertension	40%	-	-
History of kidney transplant	10%	-	-
**Dialysis parameter**			
Median dialysis vintage, months	44.50	-	-
Median intradialytic weight change, kg	-1.5	-	-
Intradialytic hypotensive episodes	.44 (1.0)	-	-
**Blood values**			
Hematocrit (l/l)	.325 (.05)	-	-
Hemoglobin (g/dl)	10.6 (1.9)	-	-
Platelets (G/l)	282.3 (90.3)	-	-
Leukocytes (G/l)	7.8 (2.0)	-	-
Sodium (mmol/l)	137.3 (3.2)	-	-
Potassium (mmol/l)	4.8 (1.3)	-	-
Urea (mg/dl)	136.9 (59.0)	-	-
Creatinine (mg/dl)	9.7 (4.1)	-	-

All data shown as mean (SD), except where noted. Charlson Comorbidity Index (CCI) corrected for dialysis patients and corrected for age in the control group. CV = Cerebrovascular; py = pack years; CKD = Chronic kidney disease. Other causes include progression of CKD due to post-operative infections, reflux diseases, analgesic medication.

MRI and neuropsychological testing were applied at two time points: (t1) dialysis-free day and (t2) after dialysis (mean 2.4 hours ± 2.2 standard deviation [SD] time after dialysis session for MRI measurement). Eight of ten patients were retested within the same dialysis cycle (24-hours time period), but due to acute intercurrent illness one patient was retested within 48 hours and one patient within one month. Healthy controls were all retested within 24 hours. All participants received a comprehensive neuropsychological assessment including major cognitive domains—attention, memory, executive functions, visuospatial processing and language—, as well as screening instruments for depression and anxiety (HADS, Hospital Anxiety and Depression Scale [17]), daytime sleepiness (Epworth Sleepiness Scale (ESS)) and fatigue [18], as previously described by Costa et al, [19]. The Mini-Mental State Examination (MMSE) [20] and the Montreal Cognitive Examination (MoCA) [21, 22] were used as brief cognitive screening tools to assess global cognition. Cued and not-cued reaction times were measured through the Alertness sub-test of the Test of Attentional Performance [23]. The Trail making test (TMT) parts A and B [24] provided an index on processing speech and cognitive flexibility. We measured the span of immediate verbal recall and auditory working memory through the digit span forwards and backwards [25]. The verbal fluency task [26], with semantic and phonemic modalities assesses functions related to language production, as well as executive functions. The Stroop test [27] was used as a measure of selective attention and inhibitory control. The California Verbal Learning Test (CVLT) test [28] was used for verbal episodic memory providing measures on verbal learning, immediate and delayed recall, as well as recognition. We used the MCGCF complex figure [29] to assess visual perception and construction (figure copy), as well as visual memory (3-minutes immediate and 30-minutes delayed recall). To exclude basal language and visual perception deficits, participants completed at baseline a short-form of the Boston Naming Test [29] and the Incomplete Letters sub-test from the Visual Object Spatial Perception (VOSP) battery [30].

Given the repeated-measures nature of the study alternate-forms were used, when available, and test order and time of assessment order were controlled for (for details [19]).

A detailed medical history for all patients with the following parameters was obtained: CKD etiology, hemodialysis vintage duration (the duration in months of the hemodialysis treatment) and volume of hemodialysis, frequency of intradialytic hypotensive episodes, as well as the following routine blood work: hematocrit, hemoglobin, leukocytes, platelets, glucose, sodium, potassium, urea, and creatinine. Comorbidities were quantified using the Charlson Comorbidity Index (CCI), which predicts the ten-year mortality by weighting several comorbid conditions, either corrected for dialysis [31] or corrected for age in the healthy control subjects [32]. The presence of cardiovascular risk factors (CVRF)—diabetes, hypertension, hypercholesterolemia, smoking, body mass index (BMI)—was recorded for all participants. For patients, CVRFs were rated using the low SCORE risk charts [33]. Medication was also listed for all participants and neuroleptics, antidepressants, opioids, as well as antiparkinsonian agents were considered as psychoactive medication.

### MRI data acquisition

MR measurements were performed on a 3 Tesla Trio MR scanner at the RWTH Aachen University (Siemens Medical Systems, Erlangen, Germany). Radio frequency (RF) excitation and signal reception were achieved with a body coil and a 32-channel receive phased array head coil, respectively. Quantitative cerebral water content measurements were based on the estimation of the MR-visible proton density (PD) [14, 16, 34–40]. It is indeed well accepted that the MR-visible proton density (PD) provides a very reliable measure of free water content present in the tissue [41]. A rough estimate of the MR-visible PD was obtained using a standard multi-slice, multi-echo Gradient Recalled Echo (GRE) MRI sequence (TR = 1800ms, TE = 5.2ms and FA = 40°), below referred to as the PD scan. Subsequently, the following transformations were applied on the PD scan prior to data analysis [16, 34, 40]: i) compensation of the transmit (B_1_^+^) and receive field (B_1_^-^) inhomogeneity ii) compensation of the T_2_* decay and iii) correction of the T_1_-saturation effect iv) correction of residual nonuniformity and v) normalization of the image to the ventricular cerebrospinal fluid (CSF) signal to obtain the water molarity (mol/L) of the tissue, expressed as a percentage of pure water molarity. The steps i) to iii) necessitate the inclusion of additional MRI scans into the water content protocol, namely, a second GRE scan (TR = 500ms, TE = 5.2ms and FA = 90°) for estimating T_1_ and applying the T_1_-saturation correction, a series of four single-shot GRE-EPI scans (TE = 11ms and FA = 30°, 60°, 90°, 120°) for estimating B_1_^+^ and a set of two low-resolution GRE scans (TR = 500ms, TE = 5.2ms and FA = 40°) for obtaining B_1_^-^ (Abbas et al., 2014). Finally, a multi-echo 3D-GRE MR acquisition was performed (TR = 35ms, FA = 12°, TE_1_ = 2.3ms, ΔTE = 2.27ms, 8 echoes). It allows estimating the T_2_* decay constant and correct for the signal decay for the first echo image of the PD-scan. The PD-scan consisted of 64 transverse slices, read in two interleaved concatenations (resulting in a gap free acquisition). The readout direction was anterior-posterior and the phase field-of-view (FOV) was adjusted to contain the entire brain (typically 190 mm). The sequences for mapping T_1_, T_2_*, B_1_^+^, B_1_^-^ were adjusted to the same slice orientation and FOV as the PD-scan. Parallel imaging was employed with the generalized auto-calibrating partially parallel acquisition (GRAPPA) technique [42] with an acceleration factor of two, which allowed shortening significantly the acquisition time. The total scan time for the water mapping protocol was fourteen minutes.

In addition to the water content protocol, a high-resolution T_1_-weighted image was acquired using a magnetization-prepared rapid gradient-echo sequence (MPRAGE) with TR = 1900ms, TE = 2.52ms, TI = 900ms, FoV = 250mm, 256x256 matrix, 176 sagittal slices, slice thickness = 1mm, resulting in an acquisition time of five minutes.

### Voxel-Based Morphometry (VBM) analysis

To investigate the structural differences between HD patients and controls, the MPRAGE datasets were analyzed using the Statistical Parametric Mapping toolbox (SPM8, www.fil.ion.ucl.ac.uk/spm) and the voxel-based-morphometry toolbox (VBM8, http://dbm.neuro.uni-jena.de/vbm). Applying a standard VBM approach [43], the MPRAGE images were spatially normalized by high-dimensional warping with a standard template and segmented into gray matter (GM), white matter (WM) and cerebrospinal fluid (CSF). To correct for individual brain sizes and to allow the comparison of the absolute amount of tissue volume, voxel values were multiplied (modulated) by the non-linear component of the Jacobian determinant derived from the spatial normalization. Finally, modulated gray matter and white matter images were smoothed with a kernel of 12 mm Gaussian full width at half maximum (FWHM). Gray matter and white matter differences between patients and controls were tested using two-sample *t*-tests, including age as a nuisance covariate. Results were accessed at highest threshold of *P* < 0.05 (family wise error [FWE]-corrected) across the whole brain and by using minimum cluster size (k_E_) of 80 voxels. Coordinates are reported in the standard anatomical space developed at the Montreal Neurological Institute (MNI).

### Global and focal analyses of quantitative brain water content

To analyze cerebral water content changes within the brain, the MPRAGE and the quantitative PD and T_1_ images delivered by the quantitative water content protocol were normalized to MNI template for each individual (1mm isotropic MNI152 T_1_–weighted), using the FSL-tool FLIRT (http://www.fmrib.ox.ac.uk/fsl), with a 12-parameter affine transformation [44]. Subsequently, the MPRAGE image was segmented into gray and white matter as well as cerebrospinal fluid using SPM8 segmentation [45]. In order to efficiently suppress the influence of partial volume effects, all voxels with gray and white matter as well as cerebrospinal fluid probabilities smaller than one were set to zero [46].

Histograms of the MR parameters of interest (PD and T_1_) were computed after brain volume segmentation. In order to estimate the PD and T_1_ values in white matter and gray matter, discriminant analyses for the bivariate histogram of (PD, T_1_) were performed using the MIXMOD software package which allows performing model-based classifications of qualitative and quantitative data (http://mixmod.org) [47]. Furthermore, global differences between HD patients and healthy controls groups were assessed using non-parametric Wilcoxon rank-sum test.

For the purpose of focal sub-analyses, several ROIs were selected from the MNI standard template (probability threshold of 75% or above was used for masking) to compute the mean values and standard deviations of water content and T_1_, separately for white matter and gray matter. The following ROIs, obtained from the MNI atlas [48], Harvard-Oxford cortical and subcortical structural atlas [49–52], were used for further screening and partitioning in an exploratory manner to quantify cerebral water content: total intracranial volume, total white matter, total gray matter, frontal, temporal, parietal and occipital cortices, caudate, putamen, thalamus, globus pallidus, para- and hippocampus, corpus callosum (body), insula, brainstem, cingulum (anterior cingulate, angular cortex, posterior cingulate cortex), cerebellum, superior frontal gyrus, middle frontal gyrus, inferior frontal gyrus (pars triangularis and pars opercularis), superior temporal gyrus, middle temporal gyrus, inferior temporal gyrus, superior parietal lobule gyrus, operculum (frontal, central and parietal), precuneus, lateral occipital cortex (superior, inferior division), supracalcarine cortex and occipital pole.

In order to report water content from mixed gray and white matter brain structures (e.g., angular gyrus with 50% gray matter and 49% white matter), the atlas-based ROI masks were multiplied by the subject specific gray or white matter mask so as to report white matter or gray matter water content value for such region. This analysis concerned only structures having more than 7% gray or white matter tissues. Only significant changes in cerebral tissue water homeostasis are reported.

### Statistical analysis

Depending on the comparison and respective test assumptions, group differences based on demographic and clinical variables were reported using the independent-sample T test, Mann-Whitney or the X^2^ test. Accounting for missing data, we calculated linear mixed models with random effects for correlated data, with education and testing order as covariates, to determine group, time and interaction of group and time effects on cognitive performance and in the regional water content. The goodness-of-fit and the normality of the residuals were verified. Association measures between clinical and cognitive data (at baseline) and water mapping data were performed by computing Person’s (r) or Spearman’s [53] correlation coefficients, with a significance threshold of 0.05. Effect sizes were calculated using Cohen’s *d* test [54] (0.3 small, 0.5 medium, 0.9 to infinity large). Bonferroni correction was used accordingly to correct for multiple comparisons.

## Results

### Neuropsychological profile

Neuropsychological performance of HD patients and healthy controls, before and after dialysis, are reported in Table 2, HD patients performed consistently worse than matched healthy controls in several cognitive domains, especially in attention (phasic and intrinsic alertness, digit span) and executive functions (verbal fluency, working memory, cognitive flexibility). We found no differences between groups regarding verbal and nonverbal memory functions, nor basal language and visuospatial functioning. We found an interaction effect of dialysis on the phasic and intrinsic alertness and immediate verbal recall performance, where patients showed a decline in performance after dialysis, whereas controls showed an improvement in performance at re-test. Patients presented with higher levels of fatigue, which remained stable around the dialysis cycle. They also scored significantly higher for anxiety and total HADS score when compared to controls.

**Table 2:** Neuropsychological performance of hemodialysis patients and healthy controls on t1 and t2 time-points.

Cognitive Domain/Test	Hemodialysis Patients (n = 10)		Healthy Controls (n = 10)		
	t1	t2	t1	t2	
	Mean (SD)	Mean (SD)	Mean (SD)	Mean (SD)	Mixed Model
**Screening**					
MoCA (total score)	24.00 (2.67)	24.00 (4.0)	26.90 (1.97)	27.7 (1.25)	g**
MMSE (total score)	24.57 (10.8)	28.83 (1.17)	29.00 (.87)	28.98 (1.45)	ns
**Attention**					
Phasic Alertness (msec.)	248.99 (38.70)	286.75 (59.87)	240.00 (35.49)	228.8 (36.49)	i*
Intrinsic Alertness (msec.)	251.13 (32.19)	290.00 (54.40)	231.00 (24.89)	218.50 (26.42)	i*
**Verbal Memory**					
Digit span forwards	6.86 (1.07)	7.00 (1.55)	8.60 (1.84)	7.00 (1.55)	g*
CVLT Total learning	51.71 (5.16)	51.67 (9.67)	55.10 (10.65)	52.80 (13.05)	ns
CVLT Interference	5.14 (1.68)	4.67 (2.42)	6.50 (2.76)	6.40 (2.46)	ns
CVLT Immediate recall	10.29 (3.15)	9.17 (3.32)	12.10 (3.25)	11.70 (3.16)	i**
CVLT Delayed recall	11.00 (2.08)	10.5 (3.02)	12.60 (2.50)	12.00 (3.13)	ns
CVLT Recognition	15.57 (.79)	15.67 (.52)	15.60 (.52)	15.60 (.69)	ns
**Nonverbal Memory**					
Complex Figure Immediate recall	20.86 (6.29)	22.00 (6.36)	26.55 (6.65)	23.39 (9.28)	ns
Complex Figure delayed recall	19.33 (6.38)	21.50 (6.89)	24.39 (8.14)	23.28 (9.99)	ns
**Visuospatial functions**					
Complex Figure Copy	31.14 (2.73)	31.33 (2.81)	33.50 (2.32)	33.0 (2.28)	g*
VOSP Incomplete Letters	19.57 (.79)	-	19.00 (.83)	-	ns
**Language**					
Boston Naming Test	14.43 (.79)	-	14.90 (.32)	-	ns
**Executive Functions**					
Digit span backwards	5.86 (.90)	5.67 (1.63)	6.70 (1.16)	5.67 (1.63)	g**
Semantic verbal fluency	25.43 (7.44)	25.00 (8.69)	33.70 (6.06)	31.00 (8.86)	g**
Phonemic verbal fluency	11.57 (4.54)	12.50 (1.52)	22.60 (7.53)	22.30 (8.83)	g***
TMT A (sec.)	54.29 (30.38)	46.5 (22.6)	28.40 (8.48)	24.70 (6.95)	g*
TMT B (sec.)	141.33 (94.58)	170.5 (132.73)	60.9 (14.29)	66.10 (32.15)	g*
Stroop Reading (sec.)	34.83 (10.84)	37.00 (12.02)	31.10 (3.07)	29.70 (4.08)	ns
Stroop Naming (sec.)	60.17 (15.07)	56.00 (10.67)	49.4 (8.21)	46.70 (8.37)	ns
Stroop Interference (sec.)	115.67 (50.47)	116.17 (58.46)	82.5 (19.10)	75.10 (15.24)	ns
**HADS**					
Anxiety	5.67 (4.58)	-	1.40 (1.58)	-	ns
Depression	2.78 (2.49)	-	.70 (1.25)	-	§**
**Fatigue**	5.17 (2.40)	5.67 (1.75)	.75 (.96)	.50 (.58)	g***
**Sleepiness (ESS)**	7.00 (2.49)	-	5.90 (3.32)	-	ns

*Note*. MoCA total score corresponds to the total uncorrected score; MoCA = Montreal Cognitive Assessment, MMSE = Mini-mental State Examination, CVLT = California Verbal Learning Test, VOSP = Visual Object and Space Perception Battery, TMT = Trail Making Test, HADS = Hospital Anxiety and Depression Scale; ESS = Epworth Sleepiness Scale. ns, not significant; i, significant interaction of group and time; g, significant main effect of group; § significant Wilcoxon-Mann–Whitney U test, with Bonferroni correction for multiple testing (*p* <.01). Mixed linear model Group x Time, with education and presentation order as covariates.

* *p* <. 05

** *p* <. 01

*** *p* <. 001.

### Voxel-based morphometry (VBM) of white and gray matter

VBM analysis revealed no significant differences in gray matter and white matter volume when comparing HD patients to healthy controls, apart from one cluster of 96 voxels in the white matter of the right inferior medial temporal lobe (x = 34, y = 4, z = -36, Z = 4.78). There were no differences in gray and white matter between measurements t1 and t2. However, total brain tissue volume was 7% lower in HD patients compared to controls.

### Brain water content analysis

**Fig 1:**
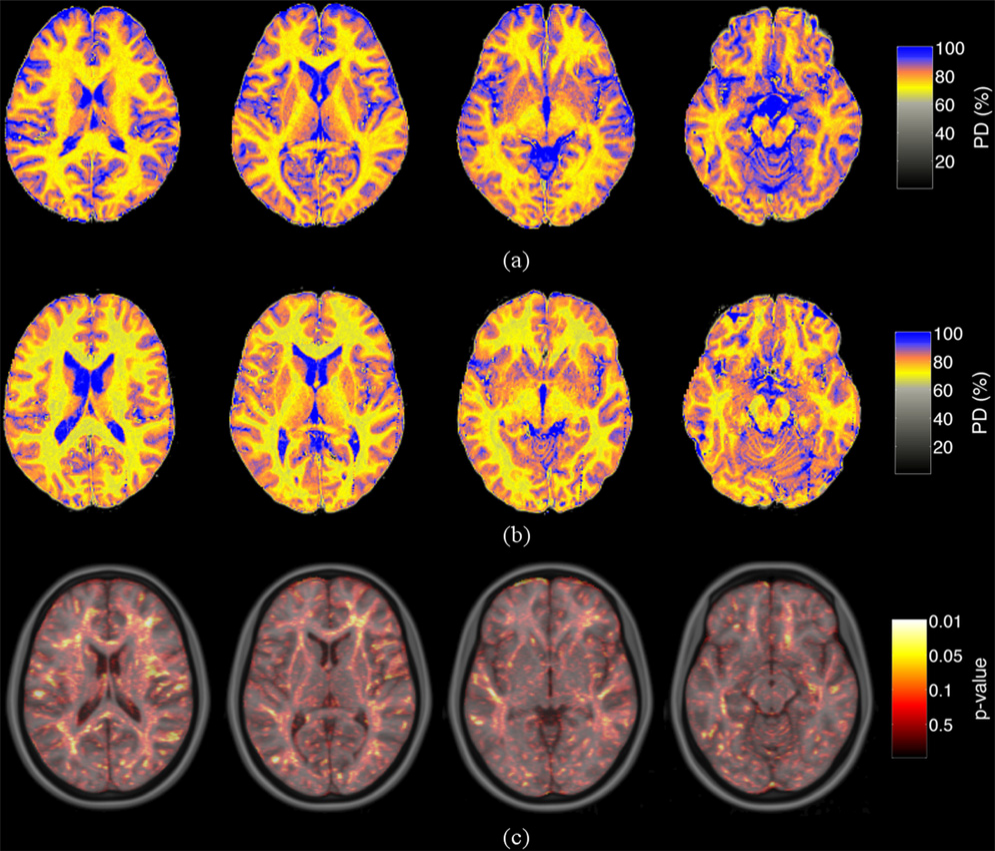
Quantitative brain water content maps. Axial slices of water content maps obtained from a representative HD patient (a) and from an age-matched healthy control (b). Differences between HD patients and healthy controls groups were assessed using non-parametric Wilcoxon rank-sum test and are shown as an overlaid p-value map on a MNI template. Analyses of water content revealed increase of water content in predominantly white matter in HD patients compared to controls. Hereby, enhanced brain hydration was found in particular in the parietal cortex, followed by occipital and fronto-temporal regions (c). The color bar in a) and b) represents the water content in percent ranging from zero to hundred percent, the color bar in c) displays the p-values.

**Fig 2:**
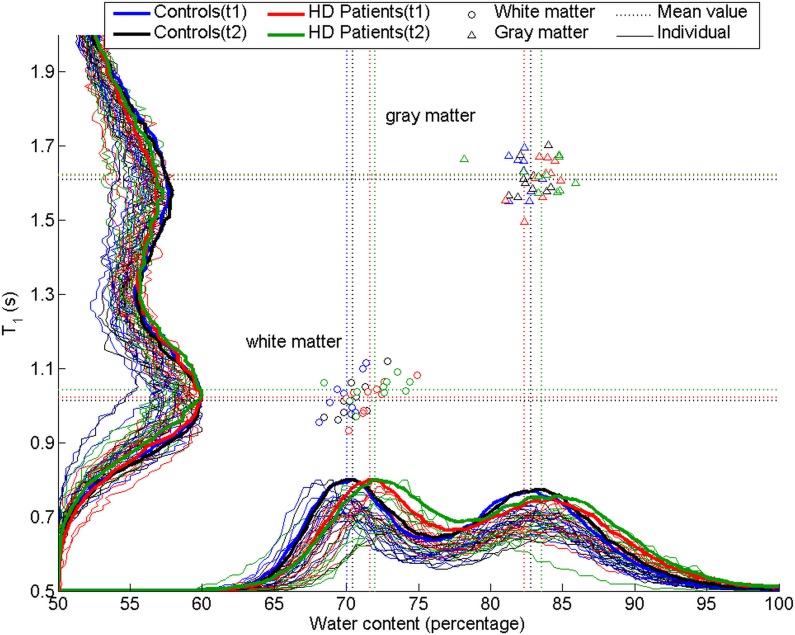
Histogram. Distributions of the brain water content (X-axis) and T1 (Y-axis) from HD patients (red thin histogram lines before dialysis, thin green histograms lines after dialysis) and healthy controls (blue thin histogram lines for first time point, black for second time point) are shown. Left: Individual data and mean value histograms (bold) for T1 (Y-axis, left) reveal no significant differences in HD patients compared to healthy controls. Lower panel: Individual data as well as mean value histograms (bold) for water content (X-axis) reveal increased white matter water content (~2%) as shown by the observed shift, but only mild changes in gray matter in HD patients compared to controls.

The quantitative water content maps (Fig 1) (an overlaid *p*-value map on a MNI template) and the histograms (Fig 2) show that the spatial distribution of the estimated brain water content was altered predominantly in the white matter (*p* = 0.03, *d* = 0.98) in HD patients as compared to controls (Table 3). Whereas increased brain water content in white matter was found in the frontal (*p* = 0.03, *d* = 0.96), temporal (*p* = 0.04, *d* = 0.90), parietal (*p* = 0.02, *d* = 1.14) and occipital (*p* = 0.03, *d* = 0.95) lobes, gray matter water content was only affected in temporal (*p* = 0.04, *d* = 0.56) and parietal lobes (*p* = 0.04, *d* = 0.94). No within-group changes in brain hydration were found for the HD and control groups before and after dialysis. Whereas, greater brain water content values in HD patients compared to controls group were found in the largest white matter bundle, the corpus callosum. Regarding the frontal lobe, increased brain water content was found in the frontal white matter (inferior frontal gyrus, operculum cortex) and gray matter regions (frontal pole, inferior frontal gyrus pars triangularis). The white matter in the temporal lobe showed increased water content in the superior temporal gyrus, middle temporal gyrus, inferior temporal gyrus and the hippocampus as well as in gray matter tissue in the superior temporal gyrus, fusiform cortex, temporal pole, and hippocampus. However, the major increase of water content alterations was revealed in the parietal lobe (large effect size), in the white matter (postcentral gyrus, parietal operculum, supramarginal gyrus, angular gyrus, intracalcarine cortex) and in the gray matter (supramarginal gyrus, angular gyrus, parietal operculum cortex, supracalcarine cortex). Increased occipital water content was found in the lateral occipital cortex and in the occipital pole. In the remaining regions the differences between HD patients and controls were not statistically significant regarding time and group using the mixed linear model.

**Table 3:** Estimated water content vales in the brain of hemodialysis patients and healthy controls on t1 and t2 time-points.

Anatomical region	Hemodialysis Patients (n = 10)		Healthy Controls (n = 10)		
	t1	t2	t1	t2	
	Mean (SD)	Mean (SD)	Mean (SD)	Mean (SD)	Mixed model
**Global water content**					
White matter	72.89 (1.61)	73.48 (2.47)	71.46 (1.06)	71.67 (1.21)	g*
Gray matter	84.42 (1.59)	84.15 (1.92)	82.93 (1.00)	83.62 (1.16)	ns
**White matter**					
Frontal lobe	72.31 (1.68)	72.98 (2.40)	70.89 (1.24)	71.14 (1.36)	g*
Temporal lobe	72.23 (1.85)	72.96 (2.54)	70.52 (1.11)	70.71 (1.19)	g*
Parietal lobe	72.36 (1.65)	72.90 (2.69)	70.97 (0.91)	71.04 (1.24)	g*
Occipital lobe	73.06 (1.91)	73.64 (2.77)	71.59 (1.17)	71.76 (1.24)	g*
Cerebellum	74.95 (1.19)	75.28 (2.41)	73.91 (0.82)	74.13 (1.09)	ns
**Gray matter**					
Frontal lobe	84.03 (1.38)	83.61 (2.07)	82.52 (1.00)	83.22 (1.39)	ns
Temporal lobe	84.14 (1.85)	84.02 (2.00)	82.38 (1.08)	82.91 (1.07)	g*
Parietal lobe	84.96 (2.27)	84.69 (2.27)	83.14 (1.31)	83.91 (1.18)	ns
Occipital lobe	84.54 (1.58)	84.05 (1.93)	83.08 (0.93)	83.89 (1.11)	i*
Cerebellum	84.77 (1.55)	84.86 (2.06)	83.73 (1.22)	84.29 (1.29)	ns
**Significant Subregions**					
Corpus callosum (body)	72.26 (2.15)	73.14 (2.82)	70.60 (1.11)	70.89 (1.22)	g*
**Frontal lobe**					
Inferior frontal gyrus pars triangularis (GM)	83.54 (1.51)	82.88 (1.89)	81.75 (1.09)	82.59 (1.47)	i*
Inferior frontal gyrus pars opercularis (WM)	72.60 (1.50)	72.86 (2.30)	71.20 (1.65)	71.10 (1.40)	g*
Frontal pole (GM)	82.79 (1.46)	82.37 (1.98)	81.47 (1.08)	82.35 (1.50)	i*
Frontal operculum cortex (WM)	72.84 (1.68)	73.39 (2.69)	70.98 (1.41)	71.40 (1.61)	g**
**Temporal lobe**					
Superior temporal gyrus posterior division (WM)	72.73 (1.67)	73.60 (3.14)	71.30 (1.60)	71.57 (1.52)	g*
Superior temporal gyrus anterior division (GM)	84.09 (2.19)	83.67 (2.25)	81.49 (1.44)	82.87 (1.49)	g*, i*
Superior temporal gyrus posterior division (GM)	84.67 (1.49)	84.01 (1.84)	82.22 (1.70)	83.35 (1.42)	g*, i*
Middle temporal gyrus temporooccipital part (WM)	73.41 (1.53)	73.94 (2.31)	71.69 (1.09)	71.91 (1.32)	g*
Inferior temporal gyrus anterior division (WM)	71.70 (2.08)	72.61 (2.67)	70.73 (1.11)	71.93 (0.97)	t**
Temporal pole (GM)	82.87 (1.39)	82.29 (2.01)	81.92 (1.18)	82.82 (1.34)	i*
Temporal fusiform cortex posterior division (GM)	84.96 (1.81)	84.44 (2.00)	82.90 (1.05)	83.76 (1.04)	g*
Hippocampus	85.83 (1.70)	85.42 (2.50)	83.79 (1.00)	84.50 (1.10)	g*
Parahippocampal gyrus anterior division (GM)	84.26 (2.07)	82.85 (2.21)	82.79 (1.49)	83.46 (1.35)	i*
Parahippocampal gyrus posterior division (WM)	74.98 (1.78)	75.41 (2.92)	73.15 (1.93)	72.93 (1.62)	g*
Central opercular cortex (WM)	73.02 (1.71)	73.63 (2.77)	71.02 (1.19)	71.18 (1.36)	g**
Central opercular cortex (GM)	84.52 (1.59)	84.47 (2.16)	82.50 (1.08)	83.20 (1.12)	g*
Insular cortex (WM)	73.26 (1.92)	74.14 (2.92)	71.89 (1.37)	71.99 (1.30)	g*
**Parietal lobe**					
Postcentral gyrus (WM)	71.74 (1.57)	72.25 (2.38)	70.42 (1.32)	70.54 (1.36)	g*
Supramarginal gyrus anterior division (WM)	72.55 (1.94)	73.13 (2.45)	71.05 (1.49)	71.03 (1.46)	g*
Supramarginal gyrus anterior division (GM)	83.32 (1.75)	83.20 (1.73)	81.69 (1.08)	82.15 (1.16)	g*
Supramarginal gyrus posterior division (WM)	72.29 (1.68)	73.19 (2.21)	70.75 (1.33)	70.87 (1.22)	g*
Supramarginal gyrus posterior division (GM)	83.88 (1.78)	83.74 (1.72)	82.01 (1.20)	82.60 (0.99)	g*
Precuneous cortex (WM)	72.52 (1.60)	73.50 (3.14)	71.24 (1.38)	71.19 (1.35)	g*
Angular gyrus (WM)	72.30 (1.59)	73.05 (2.30)	70.49 (1.08)	70.74 (1.22)	g**
Angular gyrus (GM)	84.12 (1.74)	84.07 (1.87)	82.16 (1.07)	82.69 (0.96)	g*
Parietal operculum cortex (GM)	84.33 (1.92)	84.60 (1.97)	82.42 (0.90)	83.16 (1.04)	g*
Intracalcarine cortex (WM)	72.84 (1.81)	73.60 (2.86)	71.76 (1.42)	71.44 (1.50)	g*
Supracalcarine cortex (GM)	84.61 (1.55)	84.54 (2.28)	82.83 (1.45)	83.42 (1.26)	g*
**Occipital lobe**					
Lateral occipital cortex superior division (WM)	72.00 (1.46)	72.50 (2.36)	70.21 (1.13)	70.35 (1.45)	g*
Lateral occipital cortex superior division (GM)	83.55 (1.68)	83.50 (1.95)	81.62 (1.20)	82.13 (1.12)	g*
Lateral occipital cortex inferior division (WM)	72.68 (1.68)	72.97 (2.73)	71.02 (0.83)	71.24 (1.30)	g*
Occipital pole (GM)	84.09 (2.13)	83.90 (1.86)	82.16 (1.46)	82.97 (1.43)	g*

*Note*. Mean and standard deviation values from patient and control groups, comparison of the two time points (before dialysis t1 vs. after dialysis t2). Data are given in percentage of pure water molarity; WM = white matter; GM = gray matter, remaining subregions were not significant. ns, not significant; i, significant interaction of group and time; g, significant main effect of group; t significant effect of time. Mixed linear model Group x Time.

* *p* <. 05

** *p* <. 01.

### Association with demographic, clinical parameters and cognitive performance

**Fig 3:**
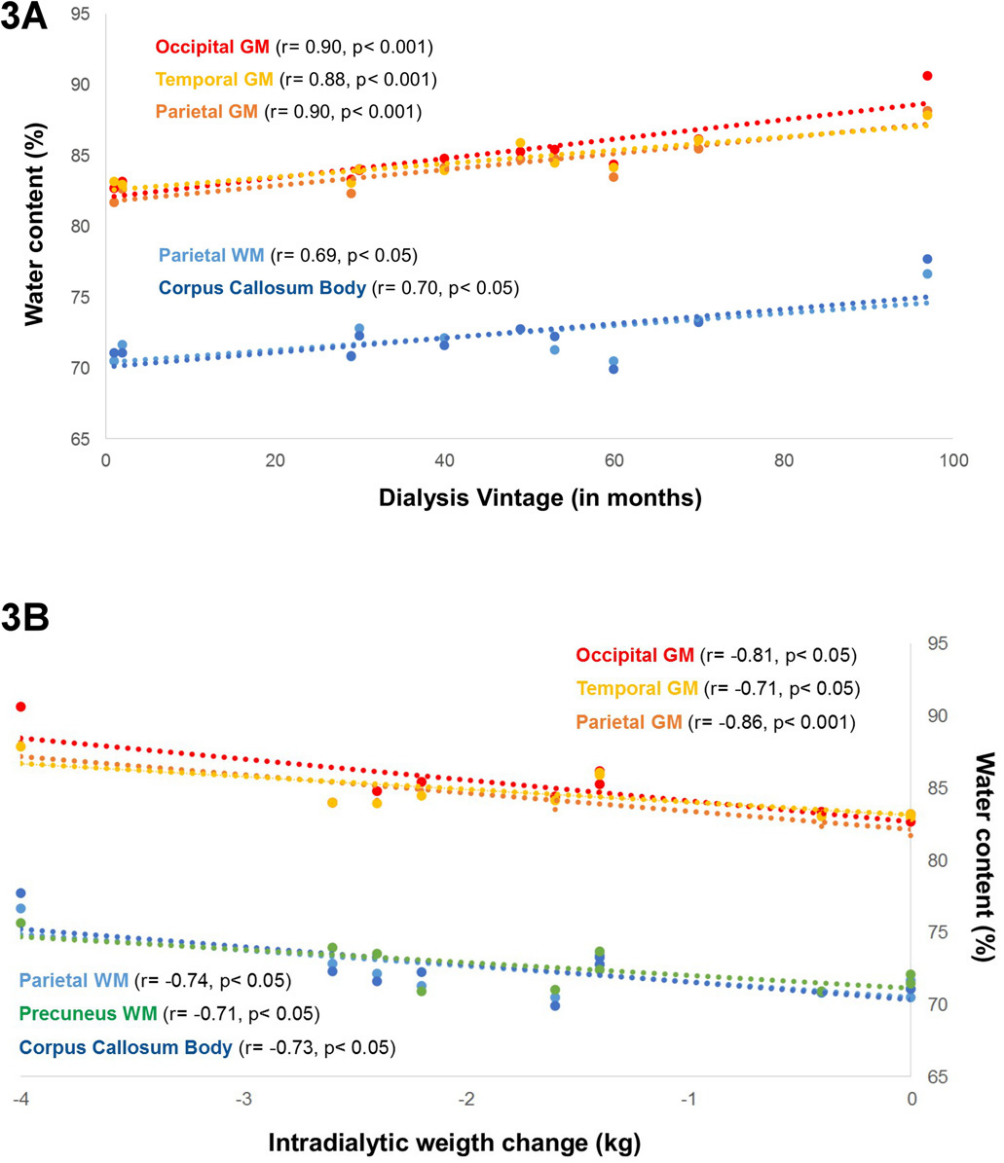
Association between brain water content and clinical parameters. Boxplots illustrating the association between water content in different focal subregions and clinical parameters. Fig 3A suggests that increased water content in several white and gray matter regions is associated with longer dialysis vintage. In Fig 3B the association between smaller intradialytic weight changes and increased water content in white and grey matter structures is shown.

Association measures showed that the duration of dialysis vintage (Fig 3A) was positively correlated with the water content observed in the parietal white matter (*r* = 0.69, *p*<0.05), in the parietal gray matter (*r* = 0.91, *p*<0.05), in the temporal gray matter (*r* = 0.89, *p*<0.05) and in the occipital gray matter (*r* = 0.88, *p*<0.05). The intradialytic weight change (3B) during the dialysis cycle was negatively correlated with parietal white matter water content (*r* = -0.74, *p*<0.01), in particular the precuneus (*r* = -0.71, *p*<0.05), occipital white matter (*r* = -0.68, *p*<0.05) and the body of the corpus callosum (*r* = -0.72, *p*<0.05). Furthermore, intradialytic weight change was negatively associated with parietal gray matter (*r* = -0.861, *p*<0.05), temporal gray matter (*r* = -0.71, *p*<0.05) and occipital gray mater water content (*r* = -0.81, *p*<0.01). Intrinsic alertness was positively correlated with frontal white matter water content (*r* = 0.73, *p*<0.05). We also found negative moderate to good correlation coefficients between the score on the anxiety and the depression scale with global white matter (*r* = -0.74, *p*<0.05), parietal white matter (*r* = -0.72, *p*<0.05) and temporal white matter water content (*r* = -0.78, *p*<0.05) as well as the water content observed in the corpus callosum (*r* = -0.69, *p*<0.05) and in the precuneus (*r* = -0.74, *p*<0.05). Remaining associations were not significant.

## Discussion

This is the first *in vivo* quantitative study based on quantitative water content estimation revealing evidence for abnormal brain water content mainly in white matter areas of cerebrum in HD patients compared to healthy controls, as most likely long-term consequences of hemodialysis. Based on current results, such changes on water content are likely to be accounted for chronic or long-term disease factors, such as dialysis vintage, and less influenced by variations during the dialysis cycle, as inferred by the lack of relevant interaction effects in our data. Although few regions showed significant interactions, with a tendency of decreased water content in HD patients after dialysis, whereas controls showed a marginal increase at re-test, similar to previous studies [55], they might be likely related to method noise or error. Furthermore, one of the major cognitive domains, altered in HD patients, namely attentional deficits, could be linked to the increased frontal white matter water content.

Water in the human brain is located in distinctive microenvironments such as axons, neurons, glial cells, myelin sheaths, extracellular space, and blood vessels. The water content measurements for gray and white matter in our control group are in very good agreement with values from available neuroimaging literature (at 1.5T and 3T), including results from invasive water content measurements in biopsy samples [14–16, 34, 36, 38, 56–59]. Changes in brain water content and/or its spatial distribution, due to cerebral or extra-cerebral mediators, can occur in both reversible and irreversible pathological conditions. Although we also revealed alterations of brain water content in the gray matter, quantitative water content maps results mainly suggest a greater vulnerability of the white matter water content in HD patients. Similarly and to greater extend, patients with hepatic encephalopathy showed an increase in cerebral white matter water content whereas water content in gray matter was globally unaffected [14], indicating an increased susceptibility of this neural tissue. Nevertheless, our results show an abnormal accumulation of excess fluid leading to early stage cerebral edema. As any cell, glia, axon and myelin sheath can be the target of toxic substances and reactive astrocytes play a significant role in cellular and tissue repair by detoxifying various noxious substances, excitotoxic aspects leading to an (cytotoxic) edema may be involved. However, the increased uptake of fluid results in local swelling, may compress the distinct microenvironments, which may finally result in dysfunctional states; and accompany various processes that damage cells, even in the early phase of degeneration. Consequently, injured cells (neurons) can shrink and disintegrate, with time degeneration, and gliosis can develop. In this context, it has to be noted that—although several neuroimaging studies in HD patients reported predominantly white matter atrophy, and to a lesser extent also in gray matter [60–66]—our sample showed only marginal changes of reduced white matter volume.

However, being largest white matter fiber bundle in the human brain, containing more than 300 million axons, the corpus callosum showed an increase of brain water content in HD patients, which was associated with longer duration of dialysis vintage und lower intradialytic weight change. Due to its highly interwoven connections the corpus callosum is integral in relaying sensory, motor, cognitive and emotional information between homologous regions in the cerebral hemispheres. Consequently, abnormalities by water diffusion in the corpus callosum may result in a broad variety of dysfunctions. The observed association between anxiety and depression related to an increase in the corpus callosum water content corroborates the previous findings of emotional dysregulation based on white matter abnormalities of the corpus callosum affecting the limbic-cortical network [67].

Major areas of hydration alterations in HD compared to controls were also found in the parietal white matter. These could be in part linked to clinical variables. Our results are in accordance with previous studies evoking the association between dialysis vintage and the presence of cognitive impairment, and white matter abnormalities [64], which reinforces the idea that longer dialysis vintage may add to an already increased vulnerability to repeated brain injury and cognitive impairment [68], through several potential pathomechanisms. One of the factors potentially involved is dialysis-related hypotension and associated changes in brain perfusion and metabolism [69]. A study that investigated the role of intradialytic hypotension in brain atrophy in chronic HD patients, established a positive relation between the incidence of hypotensive episodes during dialysis and the number of lacunae, but mainly focussed on the association between hypotension and the progression of frontal lobe atrophy over three years [62]. Although we could not establish a direct link to brain water content measures and damage to fronto-subcortical areas, which are supposed to have an increased sensitivity to cerebrovascular damage [69, 70], we hypothesize that the importance of frontal areas may be further emphasized by the link between water content in frontal areas and cognitive performance, accentuating the significance of the frontal-attentional/executive domain in the cognitive dysfunction profile of patients undergoing hemodialysis [71–73]. This neuropsychological profile of attentional and executive dysfunction was again observed in our sample, as we already described in a larger sample [19].

Furthermore, alterations in the posteromedial portion of the parietal lobe and precuneus resonate well with recent findings, that this region plays an essential role in multiple higher-order cognitive processes [74]. Moreover, they are included in the brain structures with the highest resting metabolic rates, and constitute a major part in the default mode network, which is a high level or organized default functional activity at rest [75], and which can pinpoint distinct disease-specific alterations, such as in Alzheimer’s disease [76].

Also in line with previous studies is the observation of the association between anxiety and depression and white matter, as well gray matter changes [77, 78], which might be mirrored here with the increase of water content in white matter of the temporo-parietal cortex and the corpus callosum.

This is the first *in vivo* study in HD investigating brain water content. We can state that water homeostasis is altered in HD patients, most likely as chronic effect of hemodialysis, not excluding the role of other factors. However, the results of this study and its interpretations are limited due to the selected small-sized sample. Nevertheless, it reinforces the necessity to take physiological values, as well as complementary metabolic imaging methods into account when investigating brain alterations in this population.
